# Genotyping of *Cryptosporidium* spp., *Giardia duodenalis* and *Enterocytozoon bieneusi* from sheep and goats in China

**DOI:** 10.1186/s12917-022-03447-6

**Published:** 2022-09-29

**Authors:** Penglin Wang, Ling Zheng, Linke Liu, Fuchang Yu, Yichen Jian, Rongjun Wang, Sumei Zhang, Longxian Zhang, Changshen Ning, Fuchun Jian

**Affiliations:** 1 College of Veterinary Medicine, Henan Agricultural University, No. 218 Longzihu University Area, Zhengdong New District, Zhengzhou, 450046 China; 2 International Joint Research Laboratory for Zoonotic Diseases of Henan, Zhengzhou, 450046 China

**Keywords:** *Cryptosporidium* spp., *Giardia duodenalis*, *Enterocytozoon bieneusi*, Sheep, Goats, Genotype

## Abstract

**Background:**

Few studies have molecularly characterized the potential zoonotic protozoa, *Cryptosporidium* spp., *Giardia duodenalis* and *Enterocytozoon bieneusi* in sheep and goats in China, therefore total 472 fecal samples were collected from eight provinces and infection rates of three protozoa were determined by PCR analysis of corresponding loci. All PCR positive samples were sequenced to identify the genotype.

**Results:**

The overall infection rates for *Cryptosporidium*, *G. duodenalis,* and *E. bieneusi* were 1.9% (9/472), 20.6% (97/472), and 44.5% (210/472), respectively. *C. xiaoi* (*n* = 5), *C. ubiquitum* (*n* = 3), and *C. anderson* (*n* = 1) were identified in goats. 97 *G. duodenalis* strains were successfully detected, and assembly E (*n* = 96) and assembly A (*n* = 1) were identified. Two novel *G. duodenalis* multilocus genotype (MLGs) were identified, with one belonging to subgroup AI and the other to subgroup E5. Nine known genotype (BEB6, CD6, CHC8, CHG3, CHG5, Peru6, CHG1, CHG2, and COS-I) and four new genotype (CHG26, CHG27, CHG28, and CHS18) were identified in *E. bieneusi*, with CHG3 dominant in this group.

**Conclusions:**

The present results highlight the role of sheep and goats as reservoir hosts for this three gastrointestinal pathogens. In summary, we provided a platform for more detailed research on genotyping or subtyping intestinal pathogens to better understand their risks and modes of transmission.

## Background

*Cryptosporidium*, *Giardia duodenalis*, and *Enterocytozoon bieneusi* are three opportunistic pathogens infect humans and animals. Susceptible individuals infected by these pathogens may become asymptomatic; however, other patients can experience self-limiting diarrhea or severe wasting disease, especially those immunocompromised with human immunodeficiency virus [1–3]. To date 47 *Cryptosporidium* species and approximately 70 genotypes have been identified in fish, amphibians, reptiles, birds and mammals [4]. Most *Cryptosporidium* species and genotypes are host-specific; thus far, *Cryptosporidium andersoni*, *Cryptosporidium bovis*, *Cryptosporidium ryanae*, *Cryptosporidium fayeri*, *Cryptosporidium hominis*, *Cryptosporidium ubiquitum*, *Cryptosporidium parvum*, *Cryptosporidium canis*, *Cryptosporidium scrofarum*, *Cryptosporidium suis*, and *Cryptosporidium xiaoi* have been identified in sheep and goats [5].

*Giardia duodenalis* is composed of eight assemblages: A - H, of which A and B are more common in humans, but can infect a variety of animals [1]. Assemblages C- H mainly infect non-human species. Epidemiological data on *G. duodenalis* showed that the infections with A、E assemblages were more commonly identified in sheep in China, with assemblage E being the dominant one [6].

More than 500 different *E. bieneusi* genotypes, clustering into 11 groups, have been identified based on sequence analysis of the ribosomal internal transcriptional spacer gene (ITS) [7–9]. Group 1 comprises the zoonotic evolution group containing approximately 314 genotypes, of which, genotypes A, D, EbpC and IV are the most common [10]. Group 2 genotypes were previously considered host-specific and mainly infected ruminants, but several reports indicated that group 2 genotypes such as BEB4, BEB6, I and J infected humans and other animals [8]. Thus, group 2 genotypes pose potential risks to public health whereas genotypes in groups 3–11 appear to be more host-specific.

In recent years, *Cryptosporidium*, *G. duodenalis* and *E. bieneusi* infection studies have been conducted in sheep and goats in China [6, 11–13]. However, most of these studies were limited to one region or one pathogen, thus the data were not fully comprehensive. Thereby, in order to estimate their zoonotic potential, we aimed to evaluate the molecular prevalence of *Cryptosporidium* spp., *Giardia duodenalis* and *Enterocytozoon bieneusi* infections among sheep and goats in China.

## Results

### The occurrence of *cryptosporidium*, *G. duodenalis* and *E. bieneusi*

The overall infection rates of *Cryptosporidium*, *E. bieneusi*, and *G. duodenalis* were 1.9% (9/472), 44.5% (210/472), and 20.6% (97/472), respectively. The prevalence of of *Cryptosporidium*, *G. duodenalis* and *E. bieneusi* were 2.3% (8/352), 19.3% (68/352), and 47.7% (168/352), respectively in goats. In contrast, 24.2% (29/120), and 35.0% (42/120) of sheep samples were positive for *G. duodenalis* and *E. bieneusi,* respectively (Tables 1 and 2). In addition, co-infections were detected in some samples, with the highest rate of 10.4% (49/472) observed between *E. bieneusi* and *G. duodenalis*.

**Table 1:** Infection rates and mixed infections of *Cryptosporidium*, *G. duodenalis*, and *E. bieneusi* in different regions

Region	N/T (%) of positive specimens					
	*Cryptosporidium*	*E. bieneusi*	*G. duodenalis*	*E. bieneusi+* *G. duodenalis*	*Cryptosporidium+* *E. bieneusi*	*Cryptosporidium*+ *G. duodenalis*
Henan	0.64 (1/156)	67.31 (105/156)	21.79 (34/156)	17.95 (28/156)	0.64 (1/156)	–
Llaoning	–	12.50 (2/16)	31.25 (5/16)	6.25 (1/16)	–	–
Qinghai	–	11.11 (1/9)	33.33 (3/9)	–	–	–
Gansu	–	7.70 (1/13)	46.15 (6/13)	7.69 (1/13)	–	–
Jilin	–	11.11 (2/18)	33.33 (6/18)	5.56 (1/18)	–	–
Jiangsu	**4.17 (5/120)**	47.50 (57/120)	30.00 (36/120)	14.17 (17/120)	–	2.50 (3/120)
Guizhou	–	26.42 (14/53)	9.43 (5/53)	1.89 (1/53)	–	–
Hainan	**3.45 (3/87)**	32.18 (28/87)	2.30 (2/87)	–	–	–
Total	1.91 (9/472)	44.49 (210/472)	20.55 (97/472)	10.38 (49/472)	0.64 (3/472)	–

“-“: negative; *N* Number of positive, *T* Total of analyzed samples

**Table 2:** Prevalence and genotype distribution of *Cryptosporidium*, *G. duodenalis*, and *E. bieneusi* in goats and sheep in different provinces

Species	Geographic source	No. of farms	No. (%) of positive specimens(n)			Species/assemblages/genotypes		
			*Cryptosporidium*	*E. bieneusi*	*G. duodenalis*	*Cryptosporidium*	*E. bieneusi*	*G. duodenalis*
Goats	Henan	156	0.64 (1)	66.67 (104)	21.79 (34)	***C. xiaoi*** (1)	BEB6(6)CD6(24)CHC8(1)CHG3(60)CHG5(3)peru6(1)CHG1(2)CHG2(4)CHG27(1)CHG26(1)CHG28(1)	E(34)
	Qinghai	3	–	–	–	–	–	–
	Gansu	10	–	10.00 (1)	30.00 (3)	–	CHG3(1)	E(3)
	Jiangsu	51	9.80 (5)	45.10 (23)	49.02 (25)	***C. xiaoi*** (4) *C. ubiquitm*(1)	BEB6(6)CHG1(2)CHG2(2)CHG3(11)CHG5(1)CHG28(1)	E(25)
	Hainan	87	3.45 (3)	32.18 (28)	2.30 (2)	*C. andersoni*(1) *C. ubiquitm*(2)	CHG3(16)BEB6(4)CHG5(7) CHG28(1)	E(2)
	Guizhou	45	–	26.67 (12)	8.89 (4)	–	BEB6(5)CHG1(4)CHG3(3)	E(4)
Total		352	1.91 (9)	47.73 (168)	19.32 (68)	***C. xiaoi***(5) *C. andersoni*(1) *C. ubiquitm*(3)	BEB6(21)CD6(24)CHC8(1)CHG3(90)CHG5(11)peru6(1)CHG1(8)CHG2(6)CHG26(1)CHG27(1)CHG28(3)	E(68)
Sheep	Liaoning	16	–	12.50 (2)	31.25 (5)	–	BEB6(2)	E(5)
	Qinghai	6	–	16.67 (1)	50.00 (3)	–	COS-I(1)	E(3)
	Gansu	3	–	–	100.00 (3)	–	–	E(3)
	Jilin	18	–	11.11 (2)	33.33 (6)	–	BEB6(2)	E(5) A(1)
	Jiangsu	69	–	50.72 (35)	15.94 (11)	–	BEB6(20)CHG2(2)CHG3(4)CHG5(8)CHS18(1)	E(10) A(1)
	Guizhou	8	–	25.00 (2)	13.50 (1)	–	CHG3(1)CHG5(1)	E(1)
Total		120	–	35.00 (42)	24.17 (29)	–	BEB6(24)COS-I(1)CHG2(2)CHG3(5)CHG5(9)CHS18(1)	E(28) A(1)

### Correlation analysis

As shown (Table 3), significant differences of infection rates between *E. bieneusi* and *G. duodenalis* was observed in different regions (*p* = 0.000 < 0.01), however, there are no statistically significant in the infection rates of *Cryptosporidium *were in different regions.

**Table 3:** Correlation analysis of different factors on the infection of three intestinal pathogens

Variables	No. tested	(n)No. (%) of positive specimens and 95% Cl		
		*Cryptosporidium*	*E. bieneusi*	*G. duodenalis*
Breed				
Sheep	120	**0** (0.0) -	42 (35.0) [28.7-46.]	29 (24.2) [16.4-31.9]
Goat	352	**9** (2.6) [0.7-3.8]	168 (47.7) [42.5-53.0]	68 (19.3) [15.2-23.5]
Total	472	9 (1.9) [0.7-3.1]	210 (44.5) [40.0-49.0]	97 (20.6) [16.9-24.2]
*P* value		*p* = 0.319	*p = 0.015*	*p* = 0.256
Gender				
Female	265	2 (0.8) [0.0-1.8]	115 (43.4) [37.4-49.4]	34 (12.8) [8.8-16.9]
Male	60	3 (5.5) [0.0-10.7]	35 (58.3) [45.5-71.2]	22 (36.7) [24.1-49.2]
Total	325	5 (1.5) [0.2-2.9]	150 (46.2) [40.7-51.6]	56 (17.2) [13.1-21.4]
*P* value		*p* = 0.016	*p* = 0.036	*p* = 0.000
Region				
Henan	156	1 (0.6) [0.0-1.9]	105 (67.3) [59.9-74.8]	34 (21.8) [15.2-28.3]
Liaoning	16	–	2 (12.5) [0.0-30.7]	5 (31.3) [5.7-56.8]
Qinghai	9	–	1 (11.1) [0.0-36.7]	3 (33.3) [0.0-71.8]
Gansu	13	–	1 (7.7) [0.0-24.5]	6 (46.2) [14.8-77.5]
Jilin	18	–	2 (11.1) [0.0-27.2]	6 (33.3) [9.2-57.5]
Jiangsu	120	6 (5.0) [1.0-9.0]	57 (47.5) [38.4-56.6]	36 (30.0) [21.7-38.3]
Guizhou	53	–	14 (26.4) [14.1-38.7]	5 (9.4) [1.3-17.6]
Hainan	87	2 (2.3) [0.0-5.5]	28 (32.2) [22.2-42.2]	2 (2.3) [0.0-5.5]
Total	472	9 (1.9) [0.7-3.1]	210 (44.5) [40.0-49.0]	97 (20.6) [16.9-24.2]
*P* value		*p* = 0.098	*p* = 0.000	*p* = 0.000

*p* < 0.05, the difference is significant; *P* > 0.05: no difference

*Cryptosporidium* infection rates were 0.8 and 5.5% in female and male animals (sheep and goats), respectively, indicating a significant difference (*p* = 0.016 < 0.05). Likewise, significant differences were also observed between *E. bieneusi* and *G. duodenalis* prevalence in different gender groups (*p* = 0.036 < 0.05*, p* = 0.000 < 0.01), respectively.

Also, *E. bieneusi* infection rates were 35.0 and 47.7% in sheep and goats, respectively, indicating a significant difference (*p* = 0.015 < 0.05). In contrast, no significant difference was observed in infection rates between *Cryptosporidium* and *G. duodenalis* in terms of sheep/goat breeds.

### *Cryptosporidium* species

Three *Cryptosporidium* species, *C. xiaoi* (*n* = 5), *C. ubiquitum* (*n* = 3) and *C. andersoni* (*n* = 1), were identified in goats in this study(Table 2). The *C. ubiquitum* was identified in Jiangsu(*n* = 1) and Hainan(*n* = 2), and 100% similarity with to KT922236 in lambs in Ethiopia, and KT027437 in the eastern gray squirrel in the USA. *C. xiaoi* was identified in Henan (*n* = 1) and Jiangsu(*n* = 4), which was identical to the isolate derived from goats in China (KM199748 and KM199756). In contrast, *C. andersoni* was only observed in Hainan(n = 1), and 100% similarity with HQ007049 from cattle in Brazil.

### *G. Duodenalis* assemblages and MLGs

**Fig. 1:**
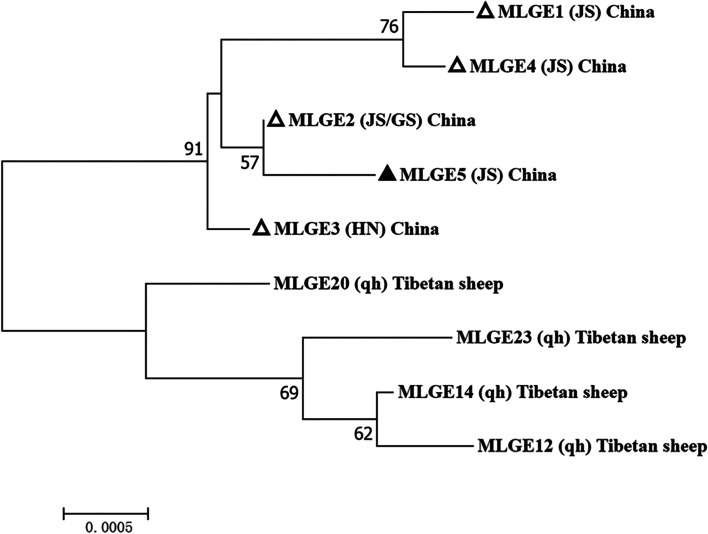
Phylogenetic relationship between *G. duodenalis* assemblage E multilocus genotype (MLG). The phylogenetic tree was constructed using a mosaic dataset of *bg, gdh* and *tpi* gene sequences, and the topology obtained by the adjacency analysis was the same. ▲: The known genotypes identified in this study. △:Reference sequences are from the studies of Jin [14]. HN: Hainan, JS: Jiangsu, GS:Gansu. qh:Qinghai

**Fig. 2:**
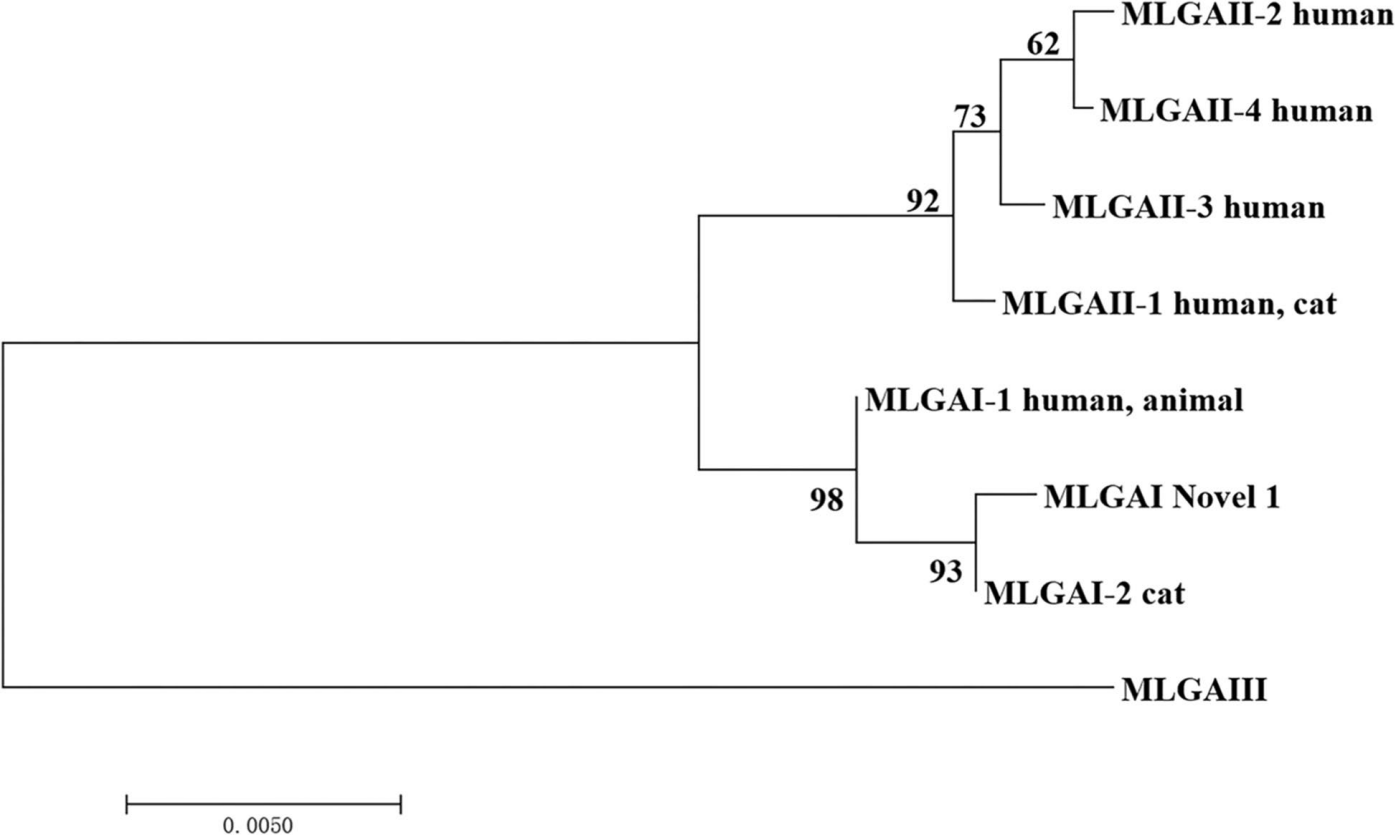
Phylogenetic relationship between *G. duodenalis* assemblage A multilocus genotype (MLG). The phylogenetic tree was constructed using a mosaic dataset of *bg, gdh* and *tpi* gene sequences, and the topology obtained by the adjacency analysis was the same. Novel 1 represent isolates from this study. Reference sequences are from the studies of Cacciò [15] and Lebbad M [16]

There are 97 PCR positive samples amplified successfully at least one gene locus (*SSU* rRNA, *bg*, *gdh*, and *tpi*) of *G. duodenalis*, 60, 22, 37, and 50 sequences of above four genes were obtained, respectively. Two *G. duodenalis* assemblages, E and A were identified in these samples (Table 2). Of the *SSU* rRNA sequences, all E assemblages belonged to the subtype E1, with their sequences showing 100% similarity to the isolate derived from cattle in China (MN593002). Of these PCR positive specimens, 11 were successfully amplified at the other three loci, and formed five assemblage E MLGs and one assemblage A MLG (Figs. 1 and 2).

### *E.bieneusi* genotypes

Based on ITS sequence analysis, a total of 13 genotypes were detected in the 210 positive samples from sheep and goats, including 9 known genotype: BEB6 (*n* = 45), CD6 (*n* = 24), CHC8 (*n* = 1), CHG3 (*n* = 96), CHG5 (*n* = 20), Peru6 (n = 1), CHG1 (*n* = 8), CHG2 (n = 8) and COS-I (n = 1), and 4 new genotype: CHG26, CHG27, CHG28, and CHS18 were detected in this study (Table 2).

**Fig. 3:**
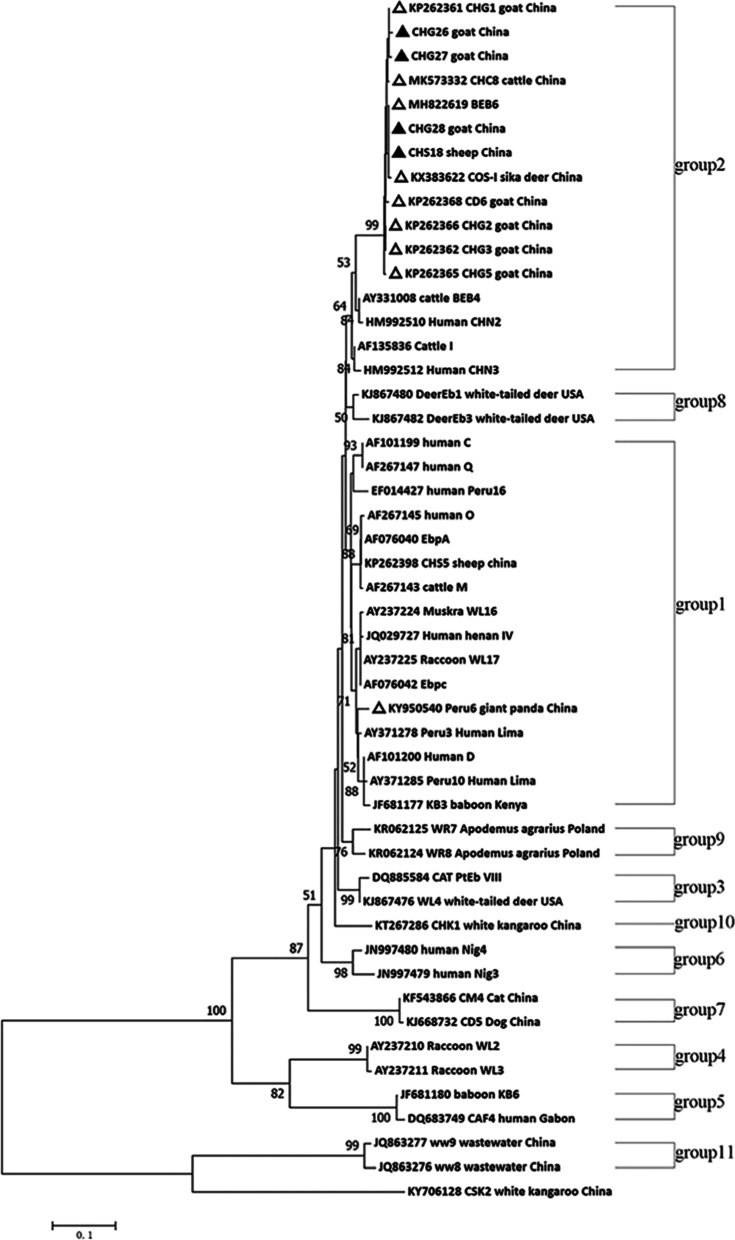
Phylogenetic analysis of *E.bieneusi* based on the ribosomal internal transcribed spacer (*ITS*) nucleotide sequence. Genotypes were based on the genetic distance calculated by the Kimura two-parameter model (Saitou and Nei, 1987), and contiguous trees were constructed using the *ITS* locus. The self-test value is 1000 repetitions. ▲: new genotype identified in this study. △: Known genotype identified in this study

The most prevalent *E. bieneusi* genotype was CHG3 (90/352, 25.6%) in goats, while BEB6 (24/120, 20.0%) in sheep. 10 out of 11 genotypes in goats detected in this study were clustered into group 2 based on phylogenetic analysis of ITS sequences and reference sequences downloaded from GenBank, while only genotype Peru 6 was belonged to 1 (Table 2, Fig. 3). In contrast, six genotypes in sheep were located in group 2.

## Discussion

In this study, *Cryptosporidium* was only detected in goats, and its prevalence (1.9%) was lower than that in Henan (34.0%) [17], Qinghai (12.3%) [18], Inner Mongolia (13.1%) [19], and Sichuan (14.6%) [20]. This low prevalence may have been due to the fact that most stool samples were collected from asymptomatic flocks. It is accepted that *Cryptosporidium* is a major opportunistic pathogen, with humans and animals with low immunity more prone to infection [21]. Additionally, the true prevalence may be underestimated, as oocyst shedding was previously reported as intermittent or below PCR detection limits [22].

*C. xiaoi*, *C. parvum*, *C. ubiquitum*, *C. andersoni*, *C. hominis* were previously documented in goats [23–27]. In this study, we detected *C. xiaoi*, *C. ubiquitum* and *C. andersoni* in goats, of which *C. xiaoi* was the dominant species. This finding agreed with other studies [17, 28]. However, goat studies conducted in Henan and Chongqing, reported that *C. andersoni* and *C. ubiquitum* were dominant species, respectively [26]. To date, many human infections caused by *C. xiaoi* and *C. ubiquitum* have been reported [29–31]. In our study, *C. andersoni* was considered a cattle-adapted species, only detected in Hainan. *C. andersoni* was first described in 2000 in the USA [32], but since then, several studies reports the parasite infects different animals [33]. Previous reports also showed that humans infected with *C. andersoni* were detected in several countries including the UK [34], Malawi [35], Australia [36], Iran [37], India and China [38]. Thus, *C. xiaoi*, *C. ubiquitum,* and *C. andersoni* are human infections and require further studies to clarify their potential zoonotic transmission in China.

When compared with *G. duodenalis* epidemiological data in other regions, the overall *G. duodenalis* infection rate in goats (19.3%) across the eight provinces was higher than that reported in Sichuan (14.9%) [11], Heilongjiang (2.9%) [39] and Anhui (6.3%) [40]. The overall *G. duodenalis* infection rate in sheep (24.2%) was higher than that reported in Henan (6.7%) [6] and Qinghai (13.1%) [14], but lower than two studies from Australia (44.0%) and Brazil (34.0%) [41–43]. The *G. duodenalis* infection rate in sheep varied greatly from region to region, however this finding agreed with previous reports showing that global *G. duodenalis* infection rates in sheep had changed dramatically from 1.5 to 55.6% [1]. The reasons for this may be due to several factors: first, samples came from different regions across China, with different climatic conditions; second, animal age information was unclear; more young animals may have been farmed in some regions; third, poor sampling technology was to blame; and fourth, insufficient management systems were in place in some farms [43].

*G. duodenalis* assemblage E was dominantly detected in goats and sheep, in agreement with several reports. Assemblage E is accompanied by strong host specificity, and mainly occurs in cloven-hoofed livestock (cattle, sheep, goats, and pigs), but also spreads between other livestock and non-human primates [13, 44]. Assemblage E was also identified in humans in Egypt, Brazil and Australia [45–47]. These observations suggested that assemblage E may lead to zoonotic infection, therefore animals infected with this assemblage could be primary hosts for animal-to-human transmission.

In this study, the only MLG belonging to assemblage A was distributed in the same branch as the MLG AI-2 isolate [15]. No human case infected with MLG AI-2 have been reported, however, further studies should be carried out to determine if it is zoonotic or not. Five assemblage E MLGs were identified in this study, and were located in different branches of the same cluster. Moreover, they were located in different clusters from assemblage E MLGs sequences from Qinghai Tibetan sheep [14] (Fig. 1). These findings suggested different geographical distributions among isolates, in agreement with previous observations in Sichuan and Xinjiang [11, 48].

The overall *E. bieneusi* infection rate was 44.5%, which was the highest infection rate among the three intestinal pathogens. The infection rate across different regions varied significantly from 0.0 to 66.7% (*p* < 0.01), and was consistent with a goat and sheep study in another parts of China [49]. The *E. bieneusi* infection rate was 35.0% in sheep, similar to that in Gansu (34.5%) [13], but higher than that in Qinghai (23.4%) [50] and Liaoning (9.4%) [49]. The *E. bieneusi* infection rate was 47.7% in goats, similar to that in Shaanxi (47.8%); lower than that in Chongqing (62.5%) [49], but higher than in Anhui (7.5%) and Yunnan (8.9%) [20].

Based on ITS sequence analysis, 13 genotypes were identified, of which the BEB6 genotype was dominant in sheep in agreement with previous reports [12, 13, 49, 51]. Other studies also reported this infection genotype was identified in cattle, cats, and geese [33]. Additionally, BEB6 was shown to infect children without diarrhea in China [8], suggesting this genotype may pose particular health threats to children. The CHG3 genotype was identified in all regions and suggested a wide geographical distribution. However, in group 2, such as genotype J, BEB4, and BEB6, were reported in human cases [52]. These data [8, 52] confirmed that genotypes in group 2 displayed zoonotic potential.

Interestingly, when analyzing parasite infection rates by sex, the rate in males were significantly higher than that in females (*p* < 0.01). To the best of our knowledge, no other study have reported these observations, therefore the infection rates of these intestinal pathogens in goats and sheep may be gender-related. To verify the accuracy of this hypothesis, the molecular epidemiology of these gastrointestinal pathogens in goats and sheep of different genders must be investigated.

## Conclusion

*Cryptosporidium*, *G. duodenalis* and *E. bieneusi* infection rates varied across different provinces, with prevalence possibly related to sex. *C. xiaoi*, *G. duodenalis* assemblage E, and *E. bieneusi* BEB6 and CHG3 were the predominant zoonotic species/assemblages/genotypes identified in this study, with important roles in pathogen transmission from animals to humans. Based on MLG analysis, *G. duodenalis* may be geographically isolated in different regions. In summary, we provided a platform for more detailed research on genotyping or subtyping intestinal pathogens to better understand their risks and modes of transmission.

## Methods

### Sample collection

**Fig. 4:**
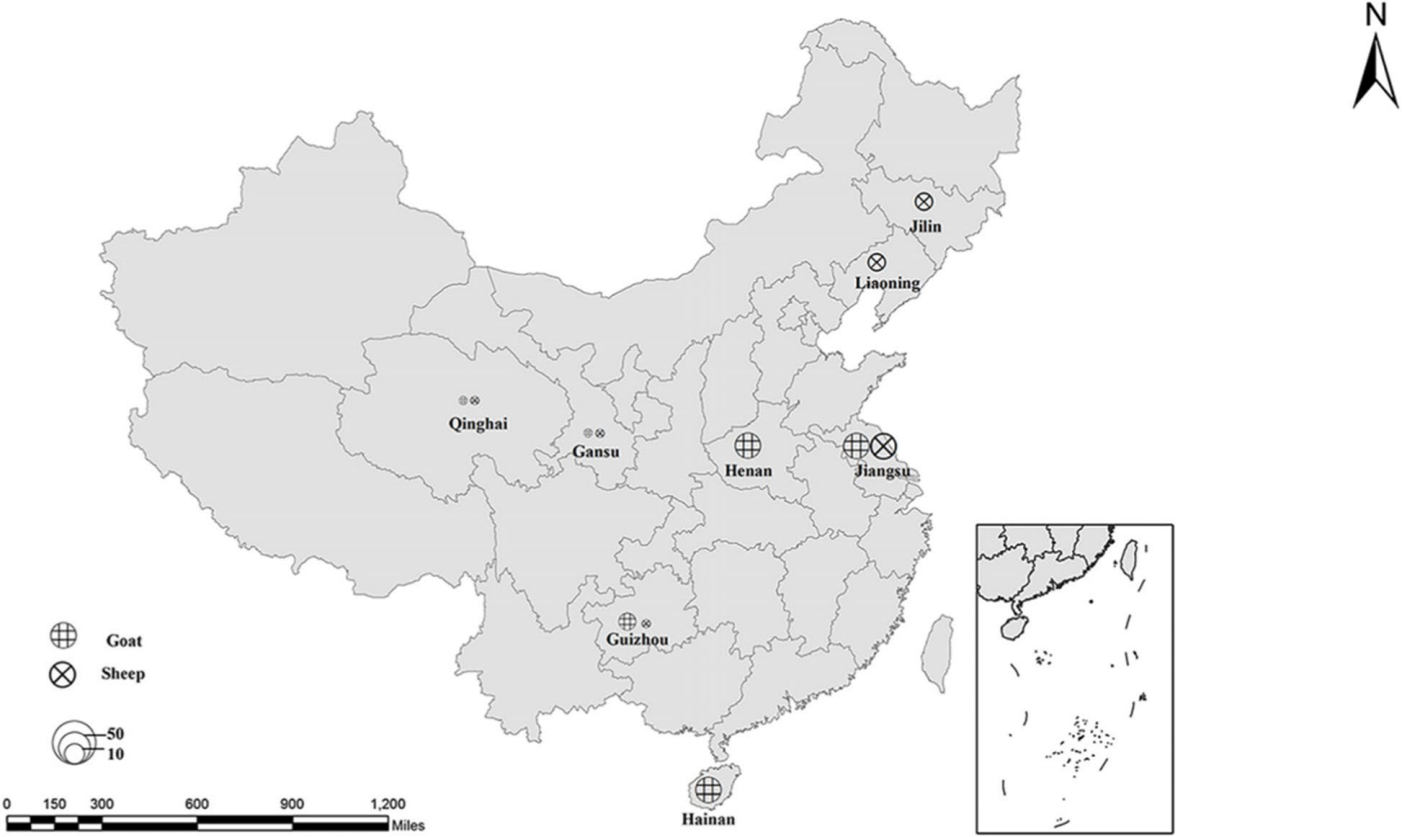
Distribution of sampling locations in China. The figure was designed by Arcgis 10.2, and the original vector diagram imported in Arcgis was adapted from Natural Earth (http://www.naturalearthdata.com)

From April to August 2019, 472 fecal samples were collected from 352 randomly selected goats and 120 randomly selected sheep in eight provinces across China (Fig. 4). In these areas, the majority of farms used the captive feeding model (e.g. Henan, Hainan, Guizhou, Gansu, Jilin, and Jiangsu), one used the grazing (Liaoning), and one used semi-grazing/semi-stable feeding (Qinghai). These farms produced sheep of all ages in good sanitary conditions. (age analysis was not conducted in this study).. For each specimen, approximately 20 g freshly voided feces was opportunistically collected using sterile latex gloves and placed into clean plastic containers on ice in a cold box. Samples were transported to the International Joint Research Laboratory for Zoonotic Diseases of Henan, China, and stored in 2.5% potassium dichromate solution at 4 °C for later use. At the time of feces collection, no diarrhea was observed in animals.

### DNA extraction and PCR amplification

Approximately 200 mg fecal samples were used to extract DNA using the E.Z.N.A®. Stool DNA Kit (Omega Biotek, Norcross, GA，USA) according to manufacturer’s instructions. Extracted DNA samples were stored at − 20 °C until required.

The small subunit (*SSU*) rRNA gene was used to screen *Cryptosporidium* samples by nested PCR amplification [53]. The *SSU* rRNA, β-giardin (*bg*), triose phosphate isomerase (*tpi*), and glutamate dehydrogenase *(gdh*) genes were used to identify *G. duodenalis* samples [15, 54–56]. *E. bieneusi* samples were determined using ITS [57]. The amplification was performed in 25 μL reaction mixtures. Positive and negative controls were included (positive samples of three protozoa, and double distilled water was used as the negative control). All PCR products were analyzed using 1% (w/v) agarose gels stained with DNA Green (Tiandz, Inc., Beijing, China) and visualized with a fluorescence gel documentation system (ZOMANBIO, Beijing, China).

### Sequence analysis

All positive amplification products were bidirectionally sequenced on an ABI PRISM™ 3730xl DNA Analyzer using the BigDye Terminator v3.1 Cycle Sequencing Kit (Applied Biosystems, Foster City, CA, USA), and all PCR positive samples were sequenced in both directions. To determine *Cryptosporidium*, *G. duodenalis* and *E. bieneusi*, genotypes, sequences were identified using reference sequences downloaded from GenBank (http://blast.ncbi.nlm.nih.gov) using Clustal X 2.1 (http://www.clustal.org/). To evaluate multilocus genotypes (MLGs) of *G. duodenalis*, we only included specimens that were successfully subtyped at all three loci, whereas ambiguous sequences (double peaks) were not included for phylogenetic analyses. Sequences were concatenated for each positive isolate to form a multilocus sequence (*bg* + *tpi* + *gdh*). Phylogenetic analyses were performed using the neighbor-joining method in MEGA 7.0 (http://www.megasoftware.net) using distance matrices calculated in the Kimura 2 parameter model. Tree reliability was evaluated using a bootstrap analysis with 1000 repetitions.

### Statistical analysis

All statistical analyses were performed using IBM SPSS Statistics Software (http://www.ibm.com/products/spssstatistics). The prevalence with the 95% confidence intervals (CI), was also calculated. Differences in corresponding infection rates among locations, breed, and gender were examined by Fisher’s exact test, and differences were considered significant at *P* ≤ 0.05.

### Nucleotide sequence accession number

Representative nucleic acid sequences reported in this paper have been submitted to NCBI’s GenBank database under the accession numbers MN845610-MN845626 and MN833262-MN833285.

## Data Availability

All of the data used or analyzed during this study are available from the corresponding author on reasonable request. Representative nucleic acid sequences reported in this paper have been submitted to NCBI’s GenBank database under the accession numbers MN845610-MN845626 and MN833262-MN833285.

## Abbreviations

*G. duodenalis*: *.*
*E. bieneusi*: *.*
*C. xiaoi*: *.*
*C. ubiquitum*: *.*
*C. Anderson*: *.*
*C. parvum*: *.*
*C. hominis*: *.*
SSU rRNA bg: *.*
gdh: *.*
tpi: *.*
ITS: *.*
MLG: *.*
